# Retraction: ‘TNF-α-induced NF-κB activation upregulates microRNA-150-3p and inhibits osteogenesis of mesenchymal stem cells by targeting β-catenin’ (2016), by Wang *et al.*

**DOI:** 10.1098/rsob.230401

**Published:** 2023-11-03

**Authors:** Open Biology editorial team

**Keywords:** mesenchymal stem cells, β-catenin, TNF-ɑ, microRNA, osteogenesis

*Open Biol.* **6**, 150258 (Published online 1 March 2016). (https://dx.doi.org/10.1098/rsob.150258)

Following an investigation, we have found that the published manuscript has substantial overlap with published manuscripts [1,2] in the text and figures (notably fig. 2*f* ‘hBM-MSCs were transfected with either miR-NC or miR-150-3p inhibitor (anti-miR), and treated with either PBS (−) or TNF-α (+), followed by Western blot analysis to examined β-catenin protein levels’ has overlap with fig.6*b* [1], and fig. 3*e* ‘Levels of phosphorylated NF-κB at Ser536 (p-NF-κB) in hBM-MSCs treated with either PBS (as control) or TNF-α’ overlap with fig. 2*b* [2]). This paper has been identified among of a set of papers that present significant text and data duplication, and therefore the data reported in this article are unreliable.

The authors have not been able to supply the raw data as requested, thus, the editors consider the conclusions of this article to be invalid. The authors have not responded to correspondence about this retraction.

Jonathon Pines FRS

Editor-in-Chief, *Open Biology*.
